# Impact of HLA-B leader matching on clinical outcomes of haploidentical hematopoietic stem cell transplantation in adult patients with acute leukemia, a retrospective cohort study

**DOI:** 10.1097/MS9.0000000000005159

**Published:** 2026-07-03

**Authors:** Nafeh Noorbakhsh, Tahereh Rostami, Soroush Rad, Amir Kasaeian, Mohammad Reza Ostadali

**Affiliations:** a Department of Hematology and Oncology, Hematologic Malignancies Research Center, Research Institute for Oncology, Hematology and Cell Therapy, Shariati Hospital, Tehran University of Medical Sciences, Tehran, Iran; b Department of Hematology and Oncology, Hematology, Oncology and Stem Cell Transplantation Research Center, Research Institute for Oncology, Hematology and Cell Therapy, Shariati Hospital, Tehran University of Medical Sciences, Tehran, Iran; c Department of Medicine, Digestive Diseases Research Center, Digestive Diseases Research Institute, Shariati Hospital, Tehran University of Medical Sciences, Tehran, Iran; d Department of Medicine, Research Center for Chronic Inflammatory Diseases, Shariati Hospital, Tehran University of Medical Sciences, Tehran, Iran; e Department of Medicine, Clinical Research Development Unit, Shariati Hospital, Tehran University of Medical Sciences, Tehran, Iran; f Department of Hematology and Oncology, Cell Therapy and Hematopoietic Stem Cell Transplantation Research Center, Research Institute for Oncology, Hematology and Cell Therapy, Shariati Hospital, Tehran University of Medical Sciences, Tehran, Iran

**Keywords:** acute leukemia, graft-versus-host disease (GVHD), haploidentical hematopoietic stem cell transplantation (haplo-HSCT), HLA-B leader matching, post-transplant cyclophosphamide (PTCy)

## Abstract

**Background:**

Haploidentical hematopoietic stem cell transplantation (haplo-HSCT) with post-transplant cyclophosphamide (PTCy) has expanded donor availability for acute leukemia, yet the clinical relevance of emerging immunogenetic markers remains uncertain. This study investigated whether donor–recipient human leukocyte antigen (HLA)-B leader matching influences transplantation outcomes in adults with acute leukemia undergoing haplo-HSCT.

**Methods:**

This retrospective analytical cohort included consecutive adults (18–65 years) with acute myeloid leukemia (AML) or acute lymphoblastic leukemia (ALL) who underwent their first haplo-HSCT at one of the most prominent referral hospitals between January 2008 and December 2022. HLA-B leader status was determined by exon 1 sequencing and classified as matched or mismatched between donor and recipient. Outcomes included overall survival (OS), disease-free survival, relapse incidence (RI), non-relapse mortality (NRM), engraftment, acute and chronic graft-versus-host disease (GVHD), and GVHD-free/relapse-free survival (GRFS). Kaplan–Meier methods, Cox regression, and competing-risk models were applied.

**Results:**

A total of 140 patients were analyzed (AML 67.9% and ALL 32.1%); 106 (75.7%) were HLA-B leader matched and 34 (24.3%) mismatched, with a median follow-up of 68 months among survivors. HLA-B leader mismatching was not associated with OS [multivariable hazard ratio (HR) = 1.02, 95% confidence interval (CI): 0.65–1.60; *P* = 0.937] or with NRM (HR 1.28; *P* = 0.34), and rates of acute GVHD (42.5% vs. 50.0%; *P* = 0.441) and chronic GVHD (25.5% vs. 29.4%; *P* = 0.65) were comparable between groups. Relapse was numerically lower in the mismatched group (11.8% vs. 24.5%; HR = 0.49, 95% CI: 0.17–1.41; *P* = 0.184). In multivariable analysis, age 25–40 years (HR = 1.67, 95% CI: 1.04–2.68; *P* = 0.034) and transplantation in CR2 or beyond (HR = 1.52, 95% CI: 1.01–2.84; *P* = 0.045) independently predicted inferior OS; acute GVHD was associated with reduced relapse risk (HR = 0.37, 95% CI: 0.16–0.86; *P* = 0.021). GRFS did not differ significantly by leader status (HR = 0.85; *P* = 0.47).

**Conclusion:**

In adults with acute leukemia undergoing PTCy-based haplo-HSCT, HLA-B leader matching was not associated with survival or GVHD. However, the observed trend toward lower relapse with leader mismatching, together with the protective association between acute GVHD and relapse, suggests a potential graft-versus-leukemia signal that warrants validation in larger cohorts.

## Introduction

Rapid proliferation and poor differentiation of young white blood cells in the bone marrow are characteristics of acute leukemia, a very aggressive hematologic cancer^[^1^]^. The best treatment option for patients diagnosed with various life-threatening malignant and non-malignant disorders, especially acute leukemia, is allogeneic hematopoietic stem cell transplantation (HSCT) from a human leukocyte antigen (HLA)-matched donor^[^2,3^]^. In this method, intact stem cells are transferred from the donor to the recipient, so the patient’s hematopoietic and immune systems are rebuilt. In addition to replacing diseased cells, this process eliminates remaining malignant cells and prevents disease recurrence through the phenomenon of the graft-versus-leukemia (GVL) effect^[^4^]^.

HIGHLIGHTS

- Adult haploidentical hematopoietic stem cell transplantation (haplo-HSCT) focus: This study analyzes outcomes in a homogeneous cohort of 140 adult patients undergoing haplo-HSCT with post-transplant cyclophosphamide (PTCy)-based graft-versus-host disease (GVHD) prophylaxis.
- Human leukocyte antigen (HLA)-B leader matching assessed: Donor–recipient HLA-B leader matching was systematically evaluated as a potential immunogenetic determinant of transplant outcomes.
- No impact on survival or GVHD: HLA-B leader matching was not associated with overall survival, disease-free survival, or acute/chronic GVHD incidence.
- Exploratory relapse signal: A non-significant trend toward lower relapse incidence was observed in HLA-B leader–mismatched transplants, suggesting a possible graft-versus-leukemia effect.
- Clinical relevance clarified: Findings suggest that HLA-B leader status alone should not guide donor selection in adult haploidentical HSCT using PTCy, though further validation is warranted.

Despite the remarkable effectiveness of this method, the main challenge in its implementation is finding a donor with complete HLA compatibility. Only about one-third of patients have a fully compatible family donor, and in many cases, the use of semi-compatible donors (haploidentical donors) is necessary^[^5^]^. In recent years, haploidentical transplantation has become an effective option for the treatment of patients without a compatible donor, with the use of new methods of post-transplant immunosuppression, especially the use of post-transplant cyclophosphamide (PTCy)^[^6,7^]^. Despite these advances, genetic differences between the donor and the recipient still have a significant impact on the clinical outcomes of transplantation. The occurrence of graft-versus-host disease (GVHD), graft failure, and relapse remains the most important challenge of this method^[^8^]^.

In the meantime, one of the new areas of research is investigating the role of polymorphisms in the HLA-B gene in the leader region. The −21 position of exon 1 of this gene can encode two types of amino acids, methionine (M) or threonine (T), which affect the level of HLA-E expression and its interaction with CD94/NKG2A/NKG2C receptors on natural killer (NK) cells^[^9,10^]^. NK cells play a key role in regulating the balance between protective immune responses (GVL) and destructive inflammatory responses (GVHD); therefore, changes in this pathway can directly affect patient survival after transplantation^[^11^]^.

Recent studies have focused on the role of the HLA-B leader match/mismatch status between the donor and recipient in transplant outcomes. The results of these studies have been contradictory; some have associated leader mismatch with better survival and reduced relapse, while others have reported increased non-relapse mortality (NRM) and GVHD^[^12^]^.

These inconsistencies suggest that the effect of the HLA-B leader is likely to depend on the type of disease, the method of immunosuppression, and the genetic characteristics of the population. Therefore, in this study, we aimed to investigate the impact of HLA-B leader matching and mismatching between donor and recipient on the outcomes of haploidentical transplantation in patients with acute leukemia. Additionally, the study has been reported in line with the STROCSS criteria^[^13^]^.

## Methods

### Study design and setting

This was a retrospective, analytical cohort study designed to evaluate the impact of donor–recipient HLA-B leader compatibility, defined by the −21 methionine/threonine dimorphism, on outcomes after haploidentical hematopoietic stem cell transplantation (haplo-HSCT) in adults with acute leukemia. The study was conducted at the Hematology, Oncology, and Stem Cell Transplantation Research Center, one of the tertiary referral centers for allogeneic transplantation. All consecutive adult patients who underwent haplo-HSCT at this center between January 2008 and December 2022 were screened for eligibility. All the patients underwent haplo-HSCT using PTCy-based GVHD prophylaxis according to institutional protocols. Although minor variations in conditioning regimens and immunosuppressive agents occurred over the study period, the overall transplantation strategy remained consistent, with PTCy forming the backbone of GVHD prophylaxis. Patients received the institutional standard haplo-PTCy regimen: cyclophosphamide 50 mg/kg on days +3 and +4, followed by tacrolimus and MMF^[^14^]^.

### Patients and eligibility criteria

Eligible patients were 18–65 years of age with a confirmed diagnosis of acute myeloid leukemia (AML) or acute lymphoblastic leukemia (ALL) according to World Health Organization (WHO) criteria^[^15,16^]^. Only the first haploidentical transplants from related donors were included. Additional inclusion criteria were the availability of high-resolution HLA typing for both donor and recipient, including exon 1 of HLA-B, as well as complete clinical and laboratory data required for outcome assessment.

Patients were excluded if they had severe pre-transplant comorbidities (such as advanced cardiac, renal, or pulmonary failure, or uncontrolled autoimmune disease), received a non-haploidentical transplant (matched sibling or unrelated donor), underwent a second or subsequent allogeneic transplant, or had missing key data or loss to follow-up immediately after discharge. The required sample size was calculated to detect a 20% absolute difference in survival between HLA-B leader-matched and mismatched groups with 95% confidence and 80% power, yielding a minimum of 120 patients. Ultimately, 140 eligible patients were included, comprising 106 in the matched group and 34 in the mismatched group, with a median follow-up of 68 months among survivors.

### Data collection

Data were obtained from electronic medical records, the institutional transplant registry (RIOHCT), and the HLA laboratory database. Two independent investigators performed data entry using a predefined data collection form, and discrepancies were resolved by comparison with the original records (double-entry verification).

The variables collected included baseline and transplant characteristics (age, sex, leukemia type, disease status at transplant, cytomegalovirus serostatus, ABO/Rh compatibility, CD34⁺ cell dose, donor age, and graft source), as well as post-transplant outcomes. Outcomes of interest were overall survival (OS), disease-free survival (DFS), relapse incidence (RI), NRM, neutrophil engraftment, acute and chronic GVHD, and GVHD-free/relapse-free survival (GRFS).

### HLA-B leader genotyping and compatibility assessment

Genomic DNA was extracted from peripheral blood samples using the QIAamp DNA Mini Kit (Qiagen, Germany) according to the manufacturer’s instructions. Exon 1 of the HLA-B gene was amplified and sequenced using Sanger sequencing. Sequence data were analyzed with SeqPilot software (JSI Medical Systems, Germany) to identify the amino acid at position −21 of the HLA-B leader peptide^[^17^]^.

Each allele was classified as methionine (M) or threonine (T) at position −21, and genotypes were categorized as MM, TT, or MT. Donor–recipient pairs were then grouped as HLA-B leader matched when the leader genotype pattern was identical, and mismatched when any discordance in the −21 M/T pattern was present^[^9^]^.

### Definitions of transplant outcomes

Neutrophil engraftment was defined as the first of three consecutive days with an absolute neutrophil count greater than 0.5 × 10^9^/L. Acute GVHD was graded using modified Glucksberg criteria, and chronic GVHD was diagnosed and staged according to National Institutes of Health consensus guidelines^[^18^]^.

OS was defined as the time from transplantation to death from any cause or the last follow-up, and DFS as the time from transplantation to either relapse, death, or the last follow-up, whichever occurred first. RI was calculated using cumulative incidence methods, considering death without relapse as a competing event, and NRM was defined as death in continuous remission. GRFS was defined as survival without grade II–IV acute GVHD, chronic GVHD requiring systemic therapy, or leukemia relapse, consistent with contemporary composite HSCT endpoints.

### Statistical analysis

Descriptive statistics were used to summarize baseline characteristics. Continuous variables were expressed as medians with ranges, and categorical variables as counts and percentages. Survival curves for OS and DFS were estimated using the Kaplan–Meier method and compared between groups with the log-rank test.

Univariable and multivariable Cox proportional hazards models were used to assess the association between covariates and OS or DFS, including HLA-B leader matching status, age group, disease status at transplant (CR1 vs. CR2 or higher), leukemia subtype, acute GVHD, donor age, and engraftment. For RI and NRM, competing risk analyses were performed using the Fine–Gray subdistribution hazard model, treating relapse and non-relapse death as competing events, respectively. Variables with a *P*-value < 0.10 in univariable analyses were considered for inclusion in multivariable models, and a two-sided *P*-value < 0.05 was regarded as statistically significant. All statistical analyses were conducted using SPSS version 26.0 (IBM Corp., Armonk, NY, USA).

## Results

### Patient characteristics

A total of 140 adult patients with acute leukemia undergoing haplo-HSCT were included in this analysis. The cohort consisted of 95 patients (67.9%) diagnosed with AML and 45 patients (32.1%) with ALL. Based on HLA-B leader dimorphism, 106 patients (75.7%) were classified into the matched group, while 34 patients (24.3%) were in the mismatched group. The median age of the entire cohort was 35 years (range: 18–65 years), with the majority falling within the 25–40 age range. There was a male predominance (60%, *n* = 84). At the time of transplantation, 60 patients (42.9%) were in the first complete remission (CR1), whereas 80 patients (57.1%) were in the second complete remission (CR2) or beyond. All patients received peripheral blood stem cells as the graft source. Baseline characteristics, including donor age, sex matching, and ABO compatibility, were comparable between the HLA-B leader matched and mismatched groups (Table 1).

**Table 1:** Baseline characteristics of patients and donors stratified by HLA-B leader compatibility.

Variable	Total (n = 140)	Matched (n = 106)	Mismatched (n = 34)	P-value
Survival status				0.344
Alive	42 (30.0%)	34 (32.1%)	8 (23.5%)	
Dead	98 (70.0%)	72 (67.9%)	26 (76.5%)	
Sex match				0.488
Match	71 (50.7%)	52 (49.1%)	19 (55.9%)	
Mismatch	69 (49.3%)	54 (50.9%)	15 (44.1%)	
Patient age				0.463
<25 years	45 (32.2%)	37 (34.9%)	8 (23.5%)	
25–40 years	65 (46.4%)	47 (44.3%)	18 (52.9%)	
>40 years	30 (21.4%)	22 (20.8%)	8 (23.5%)	
Disease status				0.569
CR1	60 (42.9%)	44 (41.5%)	16 (47.1%)	
CR2 or greater	80 (57.1%)	62 (58.5%)	18 (52.9%)	
Acute GVHD				0.441
No	78 (55.7%)	61 (57.6%)	17 (50.0%)	
Yes	62 (44.3%)	45 (42.5%)	17 (50.0%)	
Relapse				0.115
No	110 (78.6%)	80 (75.5%)	30 (88.2%)	
Yes	30 (21.4%)	26 (24.5%)	4 (11.8%)	

Percentages may not total 100 due to rounding.

### Engraftment and acute GVHD

Successful neutrophil engraftment was achieved in 90% (*n* = 126) of the study population. Graft failure was a significant predictor of mortality; in the multivariable analysis, failure to engraft was associated with a hazard ratio (HR) of 9.81 [95% confidence interval (CI): 5.21–18.44; *P* < 0.001] for OS. Acute graft-versus-host disease (aGVHD) occurred in 44.3% of patients (*n* = 62). The incidence of aGVHD was not significantly different between the HLA-B leader-matched and mismatched cohorts (42.5% vs. 50.0%, respectively; *P* = 0.441). Similarly, the incidence of chronic GVHD (cGVHD) was comparable between the two groups (25.5% in matched vs. 29.4% in mismatched; *P* = 0.65) (Table 1).

### Survival outcomes

The median follow-up duration for surviving patients was 68 months. The median OS for the entire cohort was 58 months. Kaplan–Meier survival analysis revealed no statistically significant difference in OS between the HLA-B leader-matched and mismatched groups (log-rank *P* = 0.93). Although the survival curves suggested a slight advantage for the matched group in the early post-transplant period, the curves converged over time. In the univariable Cox regression analysis, HLA-B leader mismatching showed no association with OS (HR = 1.11, 95% CI: 0.71–1.74; *P* = 0.65) (Table 2).

**Table 2:** Multivariable cox regression analysis for overall survival (OS) and relapse incidence.

	Overall survival (OS)		Relapse incidence	
Variable	HR (95% CI)	P-Value	HR (95% CI)	P-value
HLA-B leader				
Matched	Ref	-	Ref	-
Mismatched	1.02 (0.65–1.60)	0.937	0.49 (0.17–1.41)	0.184
Patient age				
<25 years	Ref	-	-	-
25–40 years	1.67 (1.04–2.68)	0.034	-	-
>40 years	1.68 (0.93–3.02)	0.086	-	-
Disease status				
CR1	Ref	-	Ref	-
CR2	1.52 (1.01–2.84)	0.045	2.27 (1.02–5.05)	0.045
Acute GVHD				
No	-	-	Ref	-
Yes	-	-	0.37 (0.16–0.86)	0.021

DFS followed a similar pattern, with no significant disparity observed between the study arms (log-rank *P* = 0.95). However, disease status at transplant was a critical determinant of outcomes. Patients transplanted in CR2 or beyond exhibited significantly inferior survival outcomes compared to those in CR1. Multivariable analysis confirmed that CR2 status was an independent risk factor for both lower OS (HR = 1.52, 95% CI: 1.01–2.84; *P* = 0.045) and lower DFS (HR = 1.57, 95% CI: 1.04–2.37; *P* = 0.029). Additionally, patients in the intermediate age group (25–40 years) demonstrated a higher risk of mortality compared to younger patients (HR = 1.67; *P* = 0.034) (Table 2).

### RI and GVL effect

The overall population had a 21.4% (*n* = 30) chance of relapsing. A clinically significant trend was noted with HLA-B leader compatibility: the recurrence rate in the mismatched group was around half that of the matched group (11.8% vs. 24.5%), although this difference did not achieve statistical significance in the univariable model (HR = 0.49, 95% CI: 0.17–1.41; *P* = 0.18).

Further corroborating the importance of immune-mediated anti-leukemic action, the incidence of aGVHD was strongly associated with a lower risk of recurrence. In the multivariate analysis, individuals who developed aGVHD had a substantially lower hazard of recurrence (HR = 0.37, 95% CI: 0.16–0.86; *P* = 0.021) than those who did not. On the other hand, an advanced disease state (CR2) remained a strong predictor of leukemia recurrence (HR = 2.27; *P* = 0.045) (Table 2).

### NRM and GRFS

NRM occurred in approximately 25% of patients. HLA-B leader compatibility did not influence NRM (HR = 1.28 for mismatch; *P* = 0.34). NRM was primarily driven by clinical complications rather than immunogenetic factors. The composite endpoint of GVHD-free, relapse-free survival (GRFS) was evaluated to assess the quality of survival. There was no significant difference in GRFS between the matched and mismatched groups (HR = 0.85; *P* = 0.47). Consistent with survival data, patients in CR2 had significantly inferior GRFS (HR = 1.49; *P* = 0.043) (Table 2).

## Discussion

Our retrospective analysis of 140 patients with acute leukemia undergoing haplo-HSCT with PTCy revealed that HLA-B leader matching status (matched vs. mismatched) did not significantly influence OS, DFS, or the incidence of GVHD. These results were not in line with recent large-scale studies that suggested a survival advantage for HLA-B leader matching, suggesting that strong T-cell modulation may lessen the immunogenetic impact of the HLA-B leader dimorphism in the context of PTCy-based prevention^[^18,19^]^.

We found no difference in survival rates between HLA-B leader-matched and mismatched donors (median OS and DFS were similar; univariable Cox HR = 1.1 for mismatches, *P* = 0.65). This is consistent with Mehta *et al*, who found that B-leader mismatches resulted in significantly increased NRM and a non-significant trend toward shorter OS in a larger haplo-HSCT cohort (*n* = 412)^[^20^]^. Lin *et al* found HLA-B leader matching to be a positive prognostic factor in myeloid malignancies, but with only a minor impact on OS or DFS^[^21^]^. In contrast, Sarmiento *et al* discovered that leader mismatches in haplo donors were highly related to greater acute GVHD rates, reflecting a stronger graft-versus-host effect but no OS benefit^[^22^]^. In our data, the CIs for OS and DFS HRs were wide, reflecting limited power (our sample is smaller than Mehta’s and others), and none of the comparisons reached statistical significance (Table 1). Thus, while point estimates hint that mismatches might slightly increase mortality (as in Mehta) or shift relapse risk, the effects were not significant. Differences in study design (single-center vs. multicenter registry), patient mix, GVHD prophylaxis (ATG vs. PTCy), and follow-up duration likely explain discordant findings. In sum, our lack of significant survival differences is broadly consistent with recent literature, which similarly reports only small or non-significant HLA-B leader effects on haplo-HSCT OS.

Instead of immunogenetics, clinical variables emerged as the primary determinants of survival in our multivariate analysis. Two independent variables significantly impacted outcomes. First, patients aged 25–40 years showed a lower survival rate compared to those under 25 years (HR = 1.67; *P* = 0.034). This observation may reflect a distinct biological behavior of leukemia in young adults, characterized by greater resistance to therapy, or differences in treatment intensity and tolerability in this intermediate age cohort^[^23,24^]^. Second, transplantation in second complete remission (CR2) or beyond was a significant negative predictor for survival (HR = 1.52; *P* = 0.045). This result supports the generally accepted worldwide agreement that the most reliable prognostic factor is the disease status at the time of transplant, highlighting the crucial significance of conducting haplo-HSCT in the first remission (CR1) whenever possible^[^25,26^]^. NRM in our study was similar between groups (HR = 1.0, *P* = 0.9), aligning with Yu *et al*, who found ABO incompatibility had no significant impact on OS or NRM in 715 allogeneic transplants, even though bidirectional ABO mismatches did increase acute GVHD risk (HR = 1.49, *P* = 0.048)^[^21^]^. Interestingly, Petersdorf *et al* reported that in unrelated cord blood transplants, HLA-B leader mismatching (with an M-leader allele) actually reduced NRM (HR = 0.44, *P* = 0.009), illustrating that the leader effect can vary by graft type^[^27^]^. Our findings further support the concept that remission status at transplant remains a stronger determinant of outcome than secondary biologic variables. In line with this, Mirzania *et al* demonstrated that achieving disease control before HSCT was a major predictor of survival in refractory AML^[^14^]^.

The overall RI in our cohort was 21%. Mismatched donors tended to have fewer relapses (HR = 0.49), but this was not statistically significant. A clinically significant finding in our study was the relationship between immune alloreactivity and relapse. In the multivariate model, the occurrence of acute GVHD was significantly associated with a reduced risk of relapse (HR = 0.37; *P* = 0.021). This demonstrates a typical GVL effect, indicating that protection against leukemic recurrence is provided by a modest level of immune activation that manifests as controllable GVHD^[^28,29^]^. Mehta *et al* also noted that certain mismatches (e.g., at DRB1) were associated with lower relapse in multivariable models, whereas B-leader mismatches alone did not have a significant effect^[^20^]^. Furthermore, although the difference did not reach statistical significance in the univariate analysis (*P* = 0.18), the cumulative incidence of relapse was notably lower in the HLA-B leader mismatched group (11.8%) compared to the matched group (24.5%). This trend suggests that HLA-B leader mismatching may enhance the anti-tumor activity of donor cells, potentially via NK cell alloreactivity mediated by differential HLA-E expression. Since HLA-B leader mismatching did not increase NRM, it may offer a favorable immunologic setup for high-risk patients^[^12,30^]^.

The specific functions of PTCy may be the reason for the differences between our null results of survival and earlier studies. By removing proliferative alloreactive T cells while preserving regulatory T cells, PTCy can reduce the effect of NK cells, which are typically regulated by the HLA-B leader/HLA-E axis. Therefore, the minor benefits of leader matching shown in unrelated donor or cord blood transplantation may be obscured by the broad immunomodulatory effects of cyclophosphamide^[^19^]^.

### Limitations

Several limitations to our study should be considered. First, the retrospective design inherently limits control over confounding variables. Second, heterogeneity in conditioning regimens and GVHD prophylaxis protocols over the 15-year study period may have influenced outcomes. Third, the sample size of the mismatched group (*n* = 34) was relatively small, which may have limited the statistical power to detect modest differences in survival or to confirm the significance of the relapse reduction. Fourth, we lacked granular molecular data, such as donor KIR genotypes or functional NK cell assays, which precluded a mechanistic analysis of the observed trends. Finally, the sample size did not permit independent evaluation of specific leader genotypes (Methionine *vs.* Threonine) within homogeneous subgroups, preventing us from analyzing whether specific allele combinations drive the outcomes.

## Conclusion

In conclusion, this study demonstrates that HLA-B leader compatibility does not significantly affect survival or the risk of GVHD in haplo-HSCT with PTCy. However, the presence of an HLA-B leader mismatch was associated with a clinically significant decrease in relapse rates (approximately a 50% reduction compared to matched controls) without adversely affecting survival. Acute GVHD also has a strong protective effect against recurrence. These findings indicate that HLA-B leader mismatching may enhance GVL effects without elevating NRM. Consequently, for patients exhibiting high-risk characteristics for whom relapse prevention is critical, the selection of an HLA-B leader-mismatched donor may represent a feasible, although not optimal, approach.

## Data Availability

The datasets generated and/or analyzed during the current study are not publicly available due to institutional privacy policies but are available from the corresponding author upon reasonable request.

## References

[1] Siegel RL Miller KD Wagle NS. Cancer statistics, 2023. CA Cancer J Clin 2023;73:17–48. 36633525 10.3322/caac.21763

[2] Auepisit C Owattanapanich W Kungwankiattichai S. Advancing the pathway to allogeneic hematopoietic stem cell transplantation through a physician–nurse coordinator system in patients with acute leukemia. Sci Rep 2025;15:24089. 40617838 10.1038/s41598-025-10061-7 PMC12228810

[3] Duda K Wieczorkiewicz-kabut A Koclęga A. Allogeneic hematopoietic stem cell transplantation remains a feasible approach for elderly with acute myeloid leukemia: a 10-year experience. Ann Hematol 2023;102:1907–14. 37074379 10.1007/s00277-023-05226-1 PMC10261218

[4] Maurer K Antin JH. The graft versus leukemia effect: donor lymphocyte infusions and cellular therapy. Front Immunol 2024;15:1328858. 38558819 10.3389/fimmu.2024.1328858 PMC10978651

[5] Rocha V Locatelli F. Searching for alternative hematopoietic stem cell donors for pediatric patients. Bone Marrow Transplant 2008;41:207–14. 18084331 10.1038/sj.bmt.1705963

[6] Elmariah H Fuchs EJ. Post-transplantation cyclophosphamide to facilitate HLA-haploidentical hematopoietic cell transplantation: mechanisms and results. Semin Hematol 2019;56:183–89. 31202428 10.1053/j.seminhematol.2018.09.002 PMC9229262

[7] Ciurea SO Bayraktar UD. “No donor”? Consider a haploidentical transplant. Blood Rev 2015;29:63–70. 25307958 10.1016/j.blre.2014.09.009 PMC4379127

[8] Malki MMA Bo-subait S Logan B. Post-transplant cyclophosphamide-based graft-versus-host disease prophylaxis after mismatched unrelated donor peripheral blood stem cell transplantation. J Clin Oncol 2025;43:2772–81. 40523209 10.1200/JCO-25-00856 PMC12353616

[9] Balgansuren G Sprague M Peterson P. HLA-B leader genotypes in a clinical population. Hla 2023;102:44–51. 36929133 10.1111/tan.15022 PMC13387073

[10] Gimeno L González-lozano I Soto-ramírez MF. CD8+ T lymphocytes are sensitive to NKG2A/HLA-E licensing interaction: role in the survival of cancer patients. OncoImmunology 2021;10:1986943. 34676148 10.1080/2162402X.2021.1986943 PMC8525952

[11] Tavaf MJ Verkiani ME Hanzaii FP. Effects of immune system cells in GvHD and corresponding therapeutic strategies. Blood Res 2023;58:2–12. 36774947 10.5045/br.2023.2022192 PMC10063589

[12] Petersdorf EW Stevenson P Bengtsson M. HLA-B leader and survivorship after HLA-mismatched unrelated donor transplantation. Blood 2020;136:362–69. 32483623 10.1182/blood.2020005743 PMC7365916

[13] Agha RA Mathew G Rashid R. Revised Strengthening the reporting of cohort, cross-sectional and case-control studies in surgery (STROCSS) Guideline: an update for the age of Artificial Intelligence. Prem J Sci 2025;10:100081.

[14] Mirzania M Khajeh-mehrizi A Yavari T. Salvage therapy with etoposide and mitoxantrone in patients with refractory acute myeloid leukemia: a retrospective cohort study. Ann Med Surg 2026;88:110–18. 10.1097/MS9.0000000000004285 PMC12767975 41497074

[15] Vardiman JW Harris NL Brunning RD. The World Health Organization (WHO) classification of the myeloid neoplasms. Blood 2002;100:2292–302. 12239137 10.1182/blood-2002-04-1199

[16] Duffield AS Mullighan CG Borowitz MJ. International Consensus Classification of acute lymphoblastic leukemia/lymphoma. Virchows Arch 2023;482:11–26. 36422706 10.1007/s00428-022-03448-8 PMC10646822

[17] Sajulga R Bolon Y-T Maiers MJ. Assessment of HLA-B genetic variation with an HLA-B leader tool and implications in clinical transplantation. Blood Adv 2022;6:270–80. 34529780 10.1182/bloodadvances.2021004561 PMC8753210

[18] Fuchs EJ McCurdy SR Solomon SR. HLA informs risk predictions after haploidentical stem cell transplantation with posttransplantation cyclophosphamide. Blood 2022;139:1452–68. 34724567 10.1182/blood.2021013443 PMC8914182

[19] Reusing SB Manser AR Groeneveld-Krentz S. HLA-E expression constitutes a novel determinant for ALL disease monitoring following hematopoietic stem cell transplantation. Bone Marrow Transplant 2021;56:1723–27. 33658648 10.1038/s41409-021-01231-y PMC8263342

[20] Mehta RS Cao K Saliba RM. HLA factors versus Non-HLA factors for haploidentical donor selection. Transplant Cell Ther 2023;29:189–98. 36470579 10.1016/j.jtct.2022.11.027 PMC10125001

[21] Yu Y Murphy MK Tan V. Impact of ABO compatibility on outcomes after allogeneic hematopoietic cell transplantation (HCT): increased risk of acute GVHD with ABO bidirectional mismatch, independent of traditional and emerging GVHD prophylaxis strategies. Cytotherapy 2026;28:102005. 41719957 10.1016/j.jcyt.2025.102005

[22] Sarmiento M Diaz J Gonzalez D. HLA-B leader mismatch predicts increased GVHD risk in haploidentical transplantation: insights from a Latin American cohort. Blood Glob Hematol 2025;2:100048.

[23] Janardan SK Miller TP. Adolescents and young adults (AYAs) vs pediatric patients: survival, risks, and barriers to enrollment. Hematology 2023;2023:581–86. 38066874 10.1182/hematology.2023000507 PMC10727024

[24] Wu X Liu H Ying K. Biological age, aging clocks, and the interplay with lymphoid neoplasms: mechanisms and clinical frontiers. Lymphatics 2025;3:19. 40726806 10.3390/lymphatics3030019 PMC12290914

[25] Mohamed Ahmed AB Elagab EA Abdalla Ahmed EA. Outcomes of allogeneic hematopoietic stem cell transplantation in adult acute myeloid leukemia: a systematic review. Cureus 2025;17:e92549. 41111822 10.7759/cureus.92549 PMC12533790

[26] Østgård LSG Lund JL Nørgaard JM. Impact of allogeneic stem cell transplantation in first complete remission in acute myeloid leukemia: a national population-based cohort study. Biol Blood Marrow Transplant 2018;24:314–23. 29051022 10.1016/j.bbmt.2017.10.019

[27] Petersdorf EW Gooley T Volt F. Use of the HLA-B leader to optimize cord blood transplantation. Haematologica 2020;106:3107. 10.3324/haematol.2020.264424 PMC8634170 33121238

[28] Burk AC Apostolova P. Metabolic instruction of the graft-versus-leukemia immunity. Front Immunol 2024;15:1347492. 38500877 10.3389/fimmu.2024.1347492 PMC10944922

[29] Sweeney C., and Vyas P. The graft-versus-leukemia effect in AML. Front Oncol 2019;9:1217. 31803612 10.3389/fonc.2019.01217 PMC6877747

[30] Coupel S Moreau A Hamidou M. Expression and release of soluble HLA-E is an immunoregulatory feature of endothelial cell activation. Blood 2006;109:2806–14. 10.1182/blood-2006-06-030213 17179229

